# Effects of thermal interventions on skeletal muscle adaptations and regeneration: perspectives on epigenetics: a narrative review

**DOI:** 10.1007/s00421-024-05642-9

**Published:** 2024-11-28

**Authors:** Tom Normand-Gravier, Robert Solsona, Valentin Dablainville, Sébastien Racinais, Fabio Borrani, Henri Bernardi, Anthony M. J. Sanchez

**Affiliations:** 1 Institute of Sport Sciences, University of Lausanne, Lausanne, Switzerland; 2 UMR866, Dynamique du Muscle et Métabolisme (DMeM), INRAE, University of Montpellier, Montpellier, France; 3 Research and Scientific Support Department, Aspetar Orthopedic and Sports Medicine Hospital, 29222 Doha, Qatar; 4 Environmental Stress Unit, CREPS Montpellier—Font-Romeu, Montpellier, France; 5 Laboratoire Interdisciplinaire Performance Santé Environnement de Montagne (LIPSEM), Faculty of Sports Sciences, University of Perpignan Via Domitia, UR 4640, 7 Avenue Pierre de Coubertin, 66120 Font-Romeu, France

**Keywords:** Heat treatment, Cryotherapy, Skeletal muscle, Exercise, Resistance and endurance training, Protein synthesis

## Abstract

Recovery methods, such as thermal interventions, have been developed to promote optimal recovery and maximize long-term training adaptations. However, the beneficial effects of these recovery strategies remain a source of controversy. This narrative review aims to provide a detailed understanding of how cold and heat interventions impact long-term training adaptations. Emphasis is placed on skeletal muscle adaptations, particularly the involvement of signaling pathways regulating protein turnover, ribosome and mitochondrial biogenesis, as well as the critical role of satellite cells in promoting myofiber regeneration following atrophy. The current literature suggests that cold interventions can blunt molecular adaptations (*e.g.*, protein synthesis and satellite cell activation) and oxi-inflammatory responses after resistance exercise, resulting in diminished exercise-induced hypertrophy and lower gains in isometric strength during training protocols. Conversely, heat interventions appear promising for mitigating skeletal muscle degradation during immobilization and atrophy. Indeed, heat treatments (*e*.*g.,* passive interventions such as sauna-bathing or diathermy) can enhance protein turnover and improve the maintenance of muscle mass in atrophic conditions, although their effects on uninjured skeletal muscles in both humans and rodents remain controversial. Nonetheless, heat treatment may serve as an important tool for attenuating atrophy and preserving mitochondrial function in immobilized or injured athletes. Finally, the potential interplay between exercise, thermal interventions and epigenetics is discussed. Future studies must be encouraged to clarify how repeated thermal interventions (heat and cold) affect long-term exercise training adaptations and to determine the optimal modalities (i.e., method of application, temperature, duration, relative humidity, and timing).

## Introduction

Physical exercise generates high metabolic and mechanical stress in an intensity- and duration-dependent manner, leading to structural damage to muscle cells and heightened inflammation and soreness (Malm 2001). According to the athlete background, prolonged and/or intense exercise can induce metabolite accumulation, a decrease in glycogen content and hyperthermia, temporarily disrupting the homeostasis of the organism (Morris et al. 2005; Fiorenza et al. 2019). A single bout of exercise can cause microscopic tears and delayed-onset muscle soreness (DOMS) (Cheung et al. 2003), with mechanical strain identified as a critical contributor to muscle damage (Newham et al. 1983; Aboodarda et al. 2011). Insufficient recovery time between training sessions can lead to overtraining, accumulated fatigue and increased risk of injury (Walters et al. 2018; Méline et al. 2019; Symons et al. 2023). Exercise-induced muscle damage and/or excessive fatigue can be detrimental to performance and prolong recovery time. Therefore, various recovery strategies have been explored in recent decades, including nutritional interventions and physiological treatments like massage, active recovery, and thermal interventions (Kellmann et al. 2018). Among physiological recovery methods, thermal exposures have gained increased attention. Cryotherapy (from the Greek ‘cryo’ meaning cold) has been the most investigated in recent years and is one of the most popular methods used by athletes (Frery et al. 2023). Cryotherapy refers to different cooling strategies such as the use of ice packs (≈ 0 to 2 °C), cooling garments, phase-change materials, whole-body cryotherapy (WBC, generally ≈ −110 to −130 °C) involving extreme cold air exposure, or cold-water immersion (CWI, generally ≈ 5 to 20 °C) (Chaillou et al. 2022). Conversely, methods that can be called ‘kaumatherapy’ (from the Greek ‘kauma’ meaning heat) (Méline et al. 2017), are gaining interest. Sauna bathing (80–100 °C), hot-water immersion (HWI, 38–44 °C), microwave diathermy (muscle temperature elevated at 38–39 °C), heat/steam sheets (muscle temperature elevated at 38–39 °C), are heat interventions commonly used by athletes during rest periods (McGorm et al. 2018).

**Fig. 1:**
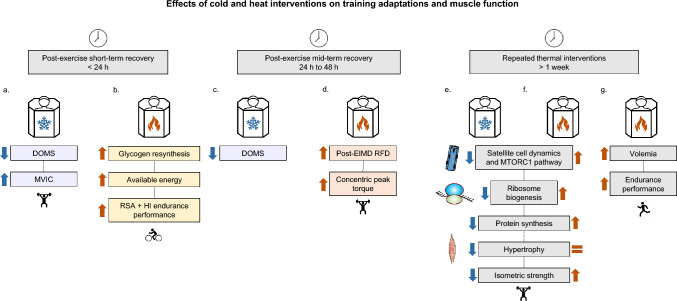
Effects of thermal interventions on training adaptations and muscle function. After a single exercise, post-exercise cold stress can decrease DOMS (delayed-onset muscle soreness) after EIMD (exercise-induced muscle damage), promoting the recovery of MVIC (maximal voluntary isometric contraction) (**a**). After a single bout of exercise, post-exercise heat intervention promotes glycogen resynthesis, accelerating the recovery of repeated sprint ability (RSA) and high-intensity (HI) endurance performance (**b**). During mid-term recovery (24–48 h), cold intervention can decrease DOMS (**c**), while heat intervention can reduce the EIMD-induced decline in the rate of force development (RFD) and concentric peak torque (**d**). Repeated exposures to cold intervention blunt chronic skeletal muscle adaptations by inhibiting the MTORC1 pathway, impairing ribosome biogenesis, and altering satellite cell dynamics, leading to a diminished protein synthesis, hypertrophy and isometric strength (**e**). In rodents, repeated exposures to heat intervention promote chronic skeletal muscle adaptations by activating the MTORC1 pathway, ribosome biogenesis, increase of satellite cell dynamics and protein synthesis (**f**). In athletes, repeated exposures to heat intervention can increase maximal isometric strength and endurance performance (**g**). However, literature does not allow to provide definitive conclusions regarding the impact of heat intervention on skeletal muscle mass and properties. Further studies are needed in humans to determine the optimal heat dose (e.g., temperature and duration) to maximize long-term training adaptations

Recent reviews have questioned the efficacy of short-term thermal interventions on recovery from exercises (Chaillou et al. 2022; Moore et al. 2022; Choo et al. 2022). Chaillou and colleagues highlighted that cold interventions might be beneficial in the early recovery phase (< 1 h post-exercise) to enhance the recovery of maximal strength (Chaillou et al. 2022) (Fig. 1a), even if the vasoconstrictor effect of cold represents a confounding factor since it could limit the cellular export of biomarkers of muscle damages. Furthermore, there are still conflicting results, as most of the studies suggest that post-exercise cryotherapy does not acutely impact the recovery of maximal strength (Pointon et al. 2011; Roberts et al. 2015a; Argus et al. 2017). The effects of cryotherapy on muscle soreness seem to stem from its analgesic properties (Meeusen and Lievens 1986). In this sense, a meta-analysis showed that CWI (5–13 °C, 10–24 min) significantly reduces DOMS 24, 48 and 96 h post-exercise (Hohenauer et al. 2015) (Fig. 1a, c). Nevertheless, a discrepancy between this subjective recovery indicator (i.e., perceived soreness) and the objective parameters (i.e., blood plasma markers, such as interleukin-6 (IL-6) or Lactate dehydrogenase) has been noted (Vaile et al. 2008). Thus, the effects of cryotherapy on objective recovery markers (e.g*.*, blood lactate, creatine kinase and cytokine levels) remain controversial and present methodological limitations (i.e., unblinded, selection bias, random sequence generation etc.) (Hohenauer et al. 2015). However, since the benefits of cryotherapy include subjective parameters, it is plausible to consider that a placebo effect may contribute to these effects, as suggested by Broatch and colleagues (Broatch et al. 2014). Furthermore, there is growing evidence that passive muscle heating may elicit several cellular adaptations. Combined with resistance exercise, passive muscle heating can increase markers of protein synthesis (Fig. 1f), such as the phosphorylation level of kinases involved in the MTORC1 (mammalian/mechanistic target of rapamycin complex 1) axis (Kakigi et al. 2011), a critical pathway regulating mRNA translation (Sanchez et al. 2019; Solsona and Sanchez 2020). Passive muscle heating increases the phosphorylation level of Akt, MTOR, 4E-BP1 (eukaryotic translation initiation factor 4E-binding protein 1) and ribosomal protein S6 (RPS6) in human skeletal muscle (Kakigi et al. 2011). These markers play a major role in growth, proliferation, and cellular survival (Sanchez et al. 2012a). However, the effects of acute HWI on other physiological variables appear inconsistent, with studies reporting contradictory findings on heart rate, muscle soreness, sprint performance, and time trial race (Kuligowski et al. 1998; Versey et al. 2013). Thus, drawing definitive conclusions about the effectiveness of acute heat intervention remains challenging, despite many studies suggesting it is beneficial. The effectiveness of thermal interventions in enhancing endurance and repeated sprint performance has been studied in twice-daily training sessions involving active individuals and elite athletes, respectively. In one study, heat accelerated recovery, whereas cold had an adverse effect after an exhaustive endurance exercise, as objectified by the mean power outputs during a second exercise session on the same day (Cheng et al. 2017). Moreover, in single muscle mice fibers, glycogen resynthesis is accelerated with heating (36 °C) (Cheng et al. 2017). The authors conclude that heating allows a faster recovery due to an increase of glycogen resynthesis rate (Fig. 1b). Consistent with these findings, repeated sprint performance was higher for active recovery and HWI compared to CWI, after high-intensity exercise training in elite short-track speed skaters (Solsona et al. 2023) (Fig. 1b). Importantly, maximal power was correlated with average muscle temperature during HWI, CWI and active recovery, and mean power was correlated with muscle temperature for active recovery and CWI (Solsona et al. 2023). This suggests a direct effect of muscle temperature modulation on exercise performance. Furthermore, it was recently found that heat and cold combination (from 3 to 45 °C) with compression (from 25 to 75 mmHg) for 10 or 20 min, improves muscle function (i.e., biomechanical changes, pain threshold, muscle strength, and tissue perfusion) in mixed martial arts athletes (Trybulski et al. 2024). These findings strongly suggests that contrast temperature interventions might be a promising tool for optimizing recovery in athletes. Several factors may contribute to the variability in the effects of thermal interventions, including the characteristics of the population studied (i.e., athletes, sedentary subjects, seniors, immobilized patients), the exercise (i.e., endurance, sprint, resistance exercise) and the therapeutic dose (i.e., timing, duration of application, treatment modality), and inter-individual variability such as variations in fat mass percentage. Due to their potentially transient nature, understanding the effects of a single thermal intervention is crucial for gaining insights into long-term adaptations. Despite the evidence concerning short intervention, the effects of the regular use of thermal interventions on long-term adaptations to exercise training remain poorly studied. It remains essential to ensure that these recovery strategies do not interfere with cellular adaptations involved in gains of physical aptitudes.

Training interventions induce both quantitative and qualitative adaptations in skeletal muscle, such as an increase in fiber size (resistance training) and enhanced cellular oxidative capacity (endurance training) (Sanchez et al. 2019). Importantly, exercise-induced cellular stress promotes physiological adaptations, leading to performance improvements (Kenttä and Hassmén 1998). Exercise training elicits a wide range of muscle adaptations, including modifications in the structural, metabolic and functional characteristics of myofibers. The phenotype of skeletal muscle is highly plastic and adapts to different modes of exercise. Eccentric exercise comprises lengthening actions and provokes sarcomere strain, notably in type II fibers (Qaisar et al. 2016). Resistance exercise particularly promotes hypertrophy through an increase in fiber cross-sectional area (CSA) and strength gains (Lopez et al. 2021). The magnitude of the increase in muscle mass can be influenced by training volume (Schoenfeld et al. 2017), although it is important to note that relying solely on training volume as a measure of training load presents limitations (Normand-Gravier et al. 2022a). For instance, high-volume resistance training leads to sarcoplasmic hypertrophy and limited strength gains (Reggiani and Schiaffino 2020). MTORC1 plays a major role in mediating hypertrophic response to resistance exercise by upregulating muscle protein synthesis rates, ribosome biogenesis, and satellite cell recruitment (Wackerhage et al. 2019; Jin et al. 2019) (Fig. 1e, f). Endurance exercise elicits different adaptations in skeletal muscle, such as a shift from type II (fast twitch fiber) to type I (slow twitch fiber) muscle fibers (Wilson et al. 2012), mitochondrial adaptations and an increase in skeletal muscle capillary density (MacInnis and Gibala 2017). Importantly, recent research has shed light on the involvement of epigenetic factors in these adaptations. Epigenetics refers to mechanisms that regulate gene expression without altering the nucleotide sequence of genes (Ehlert et al. 2013; Widmann et al. 2019). Exercise, nutrition and temperature are among the behavioral and environmental stressors that can induce epigenetic modifications (Widmann et al. 2019; Bartke and Schneider 2020). These mechanisms are critical for cellular adaptation to environmental stress (Solsona and Sanchez 2021; Solsona et al. 2022). Thus, DNA methylation, histone modifications, and expression of non-coding RNAs such as microRNAs (miRNAs, i.e., small single-stranded molecules of about 22 nucleotides) are epigenetic mechanisms that may contribute to physiological adaptations induced by exercise or thermal exposure (Sajjanar et al. 2019; Widmann et al. 2019; Normand-Gravier et al. 2022b). Specifically, histone modifications play a dynamic role in shaping the structure of chromatin, influencing cellular transcriptional capacity (Tsuchida et al. 2017). On the other hand, DNA methylation levels of promoter regions (i.e., regions located upstream of transcriptional start sites) are generally associated with gene silencing in humans (Kangaspeska et al. 2008). Although this relationship is not systematic, it is more frequently observed in promoters with (i) high CpG density, (ii) wide differentially methylated regions, or (iii) greater percentage methylation gains (de Mendoza et al. 2022). Furthermore, genes with methylation-repressed activity have a higher number of distal regulatory elements (de Mendoza et al. 2022). Nevertheless, positive correlations between promoter methylation and transcriptional changes are also found, which can be attributed to increased transcription factor affinity (Héberlé and Bardet 2019; Vizoso and van Rheenen 2021). Importantly, the methylome of human skeletal muscle presents an epigenetic memory. For instance, the differentially methylated sites remained unchanged during a detraining period of seven weeks, during which muscle mass returned to baseline (Seaborne et al. 2018). Strikingly, during a retraining period, greater hypertrophy was observed concomitantly with a greater abundance of hypomethylated genes involved in skeletal muscle growth (Seaborne et al. 2018). Recent studies have also highlighted the crucial function of miRNAs in both acute responses and long-term adaptations to exercise by impairing the translation of their target mRNAs (Solsona and Sanchez 2021; Solsona et al. 2022).

Furthermore, in recent years, there has been a growing interest in investigating the efficacy of thermal exposure to promote skeletal muscle regeneration following injury and attenuate atrophy. Atrophy can be defined as a significant loss of muscle mass and can result from immobilization during injury (Wall et al. 2013). Acutely, heating the ambient air of C2C12 cultured skeletal myotubes (47 °C for 30 min) modulated the pathways involved in protein turnover, including protein folding, pre-messenger RNA processing, messenger RNA splicing, and proteolysis (Szustakowski et al. 2007). Furthermore, the muscle atrophy F-box gene (MAFbx/Atrogin-1), an ubiquitin E3 ligase involved in proteasomal degradation, was upregulated in response to acute heat shock in mice myotubes (Szustakowski et al. 2007). Of note, these responses normalized within 24 h after acute heat stress, restoring cellular growth conditions after heat shock. The autophagy machinery, involving forkhead box class O proteins (FOXOs) and autophagic genes (Atgs), is a critical pathway for cell homeostasis and muscle integrity under stress (Sanchez et al. 2014; Møller et al. 2019). Autophagy is responsible for the degradation of proteins, lipids, polysaccharides, and intracellular organelles like damaged mitochondria (Sanchez et al. 2012b, 2014, 2015). Thus, autophagy plays a major role in skeletal muscle atrophy, particularly in the context of immobilization due to injury. In elderly individuals or patients with chronic pathologies (e.g., sarcopenia, cachexia), autophagy promotes atrophy, which can cause oxidative stress, impaired mitochondrial function, myofiber denervation, weakness, and premature mortality (Castets et al. 2013; Carnio et al. 2014; Solsona et al. 2021). Consequently, investigating the effects of thermal exposure on atrophy and skeletal muscle regeneration, with a special focus on the involvement of autophagy, has become an emerging area of research.

Hence, this narrative review aims to provide updated findings on the effects of thermal interventions on long-term exercise training adaptations for both endurance and resistance training. The second aim is to present the physiological and molecular effects of thermal interventions during skeletal muscle regeneration and discuss the potential use of thermal interventions in the context of muscle atrophy induced by immobilization. Moreover, the modulation of the autophagy pathway induced by thermal interventions during limb immobilization is detailed. Additionally, the review examines perspectives on the physiological adaptations induced by thermal interventions, particularly in relation to epigenetic modifications. Special attention will be given to the possible involvement of epigenetic factors, such as DNA methylation, in adaptation to heat intervention. Finally, the physiological effects of heat and cold interventions will be described, with a focus on the cellular responses involved in regulating skeletal muscle mass, including the role of the MTORC1 pathway in mRNA translation to proteins, ribosome biogenesis, and satellite cell recruitment.

## Effects of repeated thermal interventions on adaptations to training

### The impact of adding cold interventions to exercise training regimes

The effects of repeated cold exposures on long-term adaptations to training have been of growing interest in the last decade. A study conducted during a 14-day training camp observed a lower increase in creatine kinase (CK) activity compared to the control group in the athletes who underwent twice-daily WBC sessions (3 min at −120 °C for seven consecutive days) (Zembron-Lacny et al. 2020). However, the increase in serum myoglobin was not different between groups. On the other hand, the oxi-inflammatory mediators (i.e., the levels of hydrogen peroxide (H_2_O_2_), nitric oxide (NO), interleukin-1β, C-reactive protein, hepatocyte growth factor, insulin-like growth factor, platelet-derived growth factor and vascular endothelial growth factor) were higher in the control group. It is well-established that there is a strong correlation between accumulating reactive oxygen species (ROS) and the inflammatory response (Mittal et al. 2014). Cryotherapy may reduce this response, thereby attenuating the oxidative stress induced by exercise training. It is important to note that both inflammation and pro-oxidative imbalance are necessary for athletes to achieve adaptation (Mackey et al. 2007). A study investigating the effects of repeated WBC (3 min at −110 °C, 5 days/week for 2 weeks) on body composition found significant reductions in body fat percentage, visceral fat area and fasting glucose levels in healthy participants (Kozłowska et al. 2021). Furthermore, the study reported a reduction of circulating insulin and the homeostatic model assessment for insulin resistance score in middle-aged participants (∼ 50 years old). These improvements were partly attributed to their higher baseline values compared to their younger counterparts.

Previous studies have provided evidence that cold interventions can inhibit certain adaptations to exercise training. CWI has been shown to decrease the activity of the MTORC1 signaling pathway (Roberts et al. 2015b), which is essential for protein translation, cell growth and muscle hypertrophy (Sanchez et al. 2019). CWI also reduces ribosome biogenesis and attenuates exercise-related changes in the satellite cell pool (Roberts et al. 2015b; Figueiredo et al. 2016). On the other hand, CWI blunts and delays the increase in circulating testosterone and cytokines such as IL-6 and tumor necrosis factor-alpha (TNF-alpha) after resistance exercise (Earp et al. 2019). CWI has been shown to hamper various training adaptations following strength training, with significant effects on satellite cell activation and the activity of proteins that regulate muscle growth after a single bout of resistance exercise (Roberts et al. 2015b; Méline et al. 2017). Over a long period, CWI can lead to fewer gains in hypertrophy and completely blunt maximal isometric strength gains within a training protocol comprising 12 weeks of biweekly resistance training (Roberts et al. 2015b). In this study, CWI impeded the training-induced increase in the CSA of type II fibers, as well as the increase in the number of myonuclei per fiber. Therefore, caution must be taken when using regular cryotherapy during resistance training interventions.

Regarding endurance exercise training, results appear more contrasted. First, although CWI may increase gene expression of biogenesis mitochondrial markers (e.g., peroxisome proliferator-activated receptor-gamma coactivator-1alpha (PGC-1alpha)) after a single endurance exercise session, no effect was found in protein content of these markers on long-term studies (Broatch et al. 2018). Importantly, a six-week sprint interval training protocol with post-exercise CWI at 10 °C for 15 min was not detrimental for peak aerobic power improvement, maximal oxygen consumption (${\dot{V}O}_{2}\text{max})$, maximal uncoupled respiration of mitochondrial complexes I and II, or time trial performance (Broatch et al. 2017). A study showed that four weeks of CWI after high-intensity interval training did not alter endurance performance gains in healthy subjects (Aguiar et al. 2016). Moreover, a recent study revealed that repeated WBC (30 s at −60 °C, 150 s at −120 °C, 2 times/week, 6 weeks) did not adversely affect markers of endurance or resistance performance, such as ${\dot{V}O}_{2}\text{max}$ or muscle torque (Haq et al. 2022). However, jump height seems to be affected by WBC (Haq et al. 2022). The authors conclude that WBC does not blunt adaptations of endurance and resistance training, even if there may be an interference effect in the development of explosive power. Altogether, these findings suggests that CWI does not blunt adaptations to endurance training. On the other hand, multiple WBC sessions were found to reduce ROS and nitrogen oxide species (NOS) in blood samples after intense period of training including endurance, directed training and strength exercises in wrestle athletes (Zembron-Lacny et al. 2020), potentially leading to a reduced regenerative response. Indeed, the concentration of growth factors appeared to be negatively correlated with ROS and NOS content in the WBC group (Zembron-Lacny et al. 2020). Despite these findings, unilateral CWI blunted the gains in time to exhaustion during a single-leg incremental exercise in sedentary subjects who trained four times per week during four weeks. Furthermore, the percent change in ${\dot{V}O}_{2}\text{max}$ was significantly higher in the control leg (+ 8.2%) compared to the CWI-treated leg (−2.2%). After six weeks of training on different subjects, both groups showed a similar increase in time to exhaustion, but ${\dot{V}O}_{2}\text{max}$ and the ventilatory threshold were enhanced in the control leg only (Yamane et al. 2006). This suggest that CWI blunts adaptations to strength-endurance training.

In summary, current research suggests that cryotherapy may interfere with certain training adaptations. To date, at least five studies have provided strong evidence that repeated exposures to cold interventions following resistance exercise hinder training adaptations (i.e., hypertrophy and isometric strength) by inhibiting the MTORC1 pathway, impairing ribosome biogenesis and muscle protein synthesis rates, and altering satellite cell dynamics (Yamane et al. 2006; Roberts et al. 2015b; Figueiredo et al. 2016; Fyfe et al. 2019; Fuchs et al. 2020) (Fig. 1e). However, cryotherapy appears less detrimental to endurance training adaptations, excepted for strength-endurance where CWI appears to blunt gains in performance (Malta et al. 2021). Finally, given that literature remains elusive on the effects of CWI on endurance performance, especially in low-intensity and high-volume training, further research is needed to clarify the mixed results. The studies included in this section and treating about the effects of repeated cold interventions on adaptations to training and muscle function are summarized in Table 1.

**Table 1:** Effects of repeated cold intervention on adaptations to training and muscle function

Study	Main effects	Exercise	Population and samples	Recovery method	Effects
Yamane et al. 2006	No increase in maximal oxygen uptake and ventilatory threshold in the CWI group	ET	Healthy students	CWI	**–**
		5 min of cycling at 35% ${\dot{V}O}_{2}\text{max})$ followed by 25 min at 70% ${\dot{V}O}_{2}\text{max}$		2*20 min; 4 days/week; 4 weeks; 5 ± 1 °C	
Yamane et al. 2006	No increase in muscle endurance capacity and lower increase in maximal muscle strength	RT	Healthy students	CWI	**–**
		3 × 8 handgrip exercises at 70–80% 1RM, with 160 s of rest		20 min; 3 days/week; 4 weeks; 10 ± 1 °C	
Roberts et al. 2015a, b	Lower increase in hypertrophy and strength	Strength training	Physically active men	CWI	**–**
	Blunt the activation of satellite cells		Vastus lateralis biopsies	10 min; 2 days/week; 12 weeks; 10.1 ± 0.3 °C	
Aguiar et al. 2016	No differences in endurance performance gains compared to the control group	HIIT	Healthy subjects	CWI	∅
	No differences in protein expression compared in protein expression compared to the control group (Hsp72, AMPK, p38 MAPK)	8–12 cycling 60 s at 90–110% of peak power followed by 75 s of active recovery	Vastus lateralis biopsies	15 min; 3 days/week; 4 weeks; 10 °C	
Broatch et al. 2017	Not detrimental to endurance adaptations (peak aerobic power, maximal oxygen consumption, time trial performance)	SIT	Physically active participants	CWI	∅
		5-min cycling at 75 W, followed by 4–6 × 30-s “all-out” efforts	Vastus lateralis biopsies	15 min; 3 days/week; 6 weeks; 10 °C	
Fyfe et al. 2019	Lower increase in hypertrophy	RT	Recreationally active men	CWI	–
		Different exercises on lower and upper limbs; training intensity set at 12-RM and 120 s of recovery between each exercise	Vastus lateralis biopsies	15 min; 3 days/week; 7 weeks; 10ºC	
Fuchs et al. 2020	Reduction in myofibrillar protein synthesis rates	RT	Healthy young men	CWI	–
		4 × 8–10 repetitions on leg press and knee extension machines; training intensity set at 80% 1-RM	Vastus lateralis biopsies and blood samples	20 min; 3–4 days/week; 2 weeks; 8ºC	
Zembron-Lacny et al. 2020	Lower increase in creatine kinase activity	Training camp: endurance training (50%), directed training (24%) and special power training (26%)	Elite athletes	WBC	+
			Blood samples	Twice daily for seven consecutive days; 3 min at −120 °C	
Kozłowska et al. 2021	Decrease in body fat percentage, visceral fat area, fasting glucose and circulating insulin	Only cooling	Healthy participants	WBC 3 min; 5 days/week for 2 weeks; −110 °C	+
			Blood samples		
Haq et al. 2022	No differences in endurance and resistance performance gains compared to the control group	RT (strength and plyometric training sessions) and ET (30 min of running at 70% ${\dot{V}O}_{2}\text{max})$ )	Healthy male participants	WBC 3 min; 2 days/week for 6 weeks; 30 s at −60 °C and 150 s at −120 °C	∅
	No increase in jump height				

*1RM* one repetition maximum, *AMPK* AMP-activated protein kinase, *CWI* cold water immersion, *ET* endurance training, *HIIT* high-intensity interval training, *HSP* heat shock protein, *MAPK* mitogen-activated protein kinase, *p38* p38 mitogen-activated protein kinases, *RT* resistance training, *SIT* sprint interval training, ${\dot{V}O}_{2}\text{max})$  maximal oxygen consumption, *WBC* whole-body cryotherapy

### The impact of adding heat interventions to exercise training regimes

Recent evidence suggests that heat interventions can significantly enhance some long-term training adaptations. Regarding adaptations to endurance training, heat exposures (40 °C heated chamber, 30 min/day, 5 days/week for 3 weeks) may additionally enhance endurance-induced mitochondrial adaptations in rodents (Tamura et al. 2014). Indeed, post-exercise heat treatment increases mitochondrial enzyme activity (citrate synthase) and respiratory chain protein content in skeletal muscle, resulting in improved mitochondrial function (Tamura et al. 2014). In humans, it has been revealed that short-wave diathermy can improve mitochondrial respiratory capacity to a similar magnitude as observed with exercise (Marchant et al. 2022). However, in the latter study, only exercise significantly increased fatty acid oxidation and citrate synthase activity (Marchant et al. 2022). Repeated moderate heat exposure (warm baths, ~ 40 °C for 30 min, 5 days/week, 4 weeks) may benefit the cardiovascular system by decreasing resting sympathetic activity and heart rate, reportedly without altering baroreflex sensitivity and sympathetic responses to stress (Cui et al. 2022). Moreover, passive heat interventions (40 °C heated chamber, 40–50 min/day, 3 days/week for 6 weeks) can increase skeletal muscle capillarization and endothelial NO synthase content similarly to moderate-intensity continuous training in young sedentary males (Hesketh et al. 2019). Accordingly, previous findings from Scoon and colleagues highlighted that post-exercise sauna bathing (~ 30 min/day, ~ 4 days/week for 3 weeks, ~ 90 °C, humid sauna) may also be beneficial for increasing blood volume and endurance performance in sub-elite athletes (Scoon et al. 2007) (Fig. 1 g). The authors of this study conclude that repeated post-exercise sauna bathing produces a worthwhile improvement in endurance athletes. They report a 32% increase in the time to exhaustion test, which is estimated to correspond to a 1.9% enhancement in performance over a 5-km time trial. Altogether, these results indicate that heat interventions could be an effective approach for improving muscle function and enhancing exercise performance in sedentary population and sub-elite endurance athletes, respectively. However, a recent study failed to find effects of post-exercise dry sauna bathing (30 min, 3 times per week for 4 weeks, 89 ± 3 °C, 10 ± 2% relative humidity) on hematological adaptations in physical education students (Sitkowski et al. 2022). Indeed, no additional effects were found on hematological variables (i.e., hemoglobin mass, ferritin concentration) and exercise capacity (i.e., peak oxygen uptake, maximal power and power at gas exchange threshold) with heat interventions. This conflicting result might be explained by the differences in humidity (humid versus dry sauna). A humid sauna increases more the thermal load than a dry sauna and might explain the differences observed in hematological adaptations and exercise performance (Henderson et al. 2021). It is also suggested that a higher temperature (∼100 °C) might be necessary to have positive effects on exercise performance. In this sense, a recent study has demonstrated that dry sauna bathing at 100 ± 3 °C (40 min, 3 days/week, 3 weeks) increases physical performance (work capacity and muscle flexibility) in semi-professional football players (Bartolomé et al. 2021). Despite these findings, studies on the effects of heat interventions on training adaptations in other disciplines, particularly high-intensity sports, remain limited. It is worth noting that, in short-track speed skating, a discipline associating explosive power and complex coordination, maximal isometric strength of the knee extensors can be increased by the regular use of HWI (40.3 °C for 20 min, 4 days/week for 4 weeks) (Méline et al. 2021). In addition, a tendency was observed for improvement in ${{\dot{V}O}_{2}}_{\text{max}}$ in these elite athletes (Méline et al. 2021). However, it was also recently found that regular HWI may impair some adaptations in elite teen archers, another discipline requiring complex coordination but less mechanical and energetic constraints than short-track speed skating (Hung et al. 2018). Indeed, it was found that shooting performance and postural stability decreased after HWI (30 min at 40 °C twice a week for two weeks) (Hung et al. 2018). This result suggests that caution should be exercised when considering HWI for activities requiring high control of postural stability.

Furthermore, results appear more controversial concerning the effects of heat interventions on muscle mass and strength (Labidi et al. 2021). It was previously observed in healthy subjects that repeated and prolonged localised heat exposures (8 h/day, 4 days/week during 10 weeks) through heat- and steam-generating sheets can increase maximal isometric strength by 5.8%, muscle size by 2.7–6.1% and fiber CSA by 8.3%, without exercise training (Goto et al. 2011). Moreover, Hafen and colleagues investigated the effects of six days of localised heat interventions (pulsed shortwave diathermy, 2 h/day for 6 consecutive days) (Hafen et al. 2018). They found an increased expression of mitochondrial biogenesis markers (PGC-1alpha, METP (mitochondrial electron transport protein) complexes I and V). Hence, muscle heating seems to stimulate signaling pathways involved in muscle function (i.e., protein synthesis and mitochondrial biogenesis). Strength and muscle contractile function are also increased with repeated heat exposures (Racinais et al. 2017; Kim et al. 2020). Interestingly, there may be a negative correlation between training status and the physiological effects of heat interventions, with elite athletes deriving fewer benefits compared to sedentary or people affected by disease. A recent study investigated the effects of six weeks of localised heat interventions (heat pads, 8 h/day, 5 days/week for 6 weeks) on muscle oxidative and microvascular function in physically active participants (Ihsan et al. 2023). There were no effects on gastrocnemius aerobic function. This study supports the hypothesis that trained subjects require a more potent heat stimulus (increased duration and/or temperature) to undergo skeletal muscle adaptations. A recent study seems to corroborate this hypothesis, showing no effects of post-resistance exercise HWI on body composition and resistance performance in rugby players (Horgan et al. 2023). Moreover, the differences in muscle temperature across studies could potentially account for discrepancies in the physiological benefits observed with heat interventions. In this sense, a recent study emphasizes the need to achieve a potent stimulus by elevating muscle temperature above 39 °C to induce significant physiological adaptations (Ihsan et al. 2023). Altogether, these data highlighted that repeated heat interventions may enhance adaptations to endurance and resistance training (i.e., high-intensity endurance performance and isometric strength) when muscle temperature is sufficiently increased. Thus, it is of paramount importance to measure and ensure that the muscle temperature reaches 39 °C.

Finally, caution must be taken regarding the optimal temperature and exposure duration when using heat intervention. Indeed, a recent study compared the impact of two different HWI interventions (45 min at 40 °C versus 41 °C) immediately after exercise-induced muscle damage (EIMD, five sets of 10 maximal eccentric contractions) on core body temperature and functional markers of muscle recovery (Sautillet et al. 2023). The authors highlighted that 45 min of HWI at 41 °C, elevating core temperature to 38.5–39 °C by the end of the immersion, may be the optimal dose to improve muscle recovery in the late recovery phase (24–48 h post-exercise). Specifically, HWI at 41 °C reduced the EIMD-induced decline in the rate of force development and concentric peak torque (at 24 and 48 h, respectively) (Fig. 1d). Hence, this study shows that there is a strong dose–response relationship for heat intervention, and that a difference of 1 °C during HWI can lead to different results. Another study found that 2 h of HWI at 42 °C increases muscle temperature up to 39 °C, with no increase in systolic blood pressure. The authors concluded this protocol is safe and tolerable, and that the increase of 2.8 °C in muscle temperature corresponds to the optimal zone for promoting cellular adaptations and muscle growth (Rodrigues et al. 2020a). However, while transient heat stress may benefit skeletal muscle adaptations, prolonged heat intervention could dysregulate cellular homeostasis and become detrimental. Accordingly, a study conducted in pigs showed that 24–72 h of environmental heat stress (35 °C) significantly changed the expression of markers of phagophore formation and maturation and autophagosome degradation (UNC-51-like kinase 1 phosphorylation, Microtubule-associated protein light chain 3 A/B-II to A/B-I ratio, p62 protein expression), indicating an accumulation of autophagosomes in muscle cells (Brownstein et al. 2017). Although the autophagic flux were not directly measured, these results may reflect decreased lysosomal degradation. Of note, PINK1 and BNIP3L (PTEN‐induced putative kinase 1 and BCL2/Adenovirus E1B 19 kDa interacting protein 3‐like), two mitophagic markers, decreased with heat exposure. This would imply impaired renewal of cellular components, particularly mitochondria, raising concerns about the viability of such prolonged treatments for maintaining cellular quality and function.

In summary, improper use of thermal interventions can delay recovery and impair training adaptations, such as gains in isometric and endurance strength, and hypertrophy, as observed with the regular use of CWI (Takagi et al. 2011; Roberts et al. 2015b; Méline et al. 2017; Malta et al. 2021). Therefore, matching specific training stimuli with the appropriate use of thermal strategies is fundamental to avoid interferences and to potentially favour training adaptations. While cryotherapy has been shown to blunt certain adaptations to training, only a limited number of studies have investigated the effects of repeated heat interventions on adaptations to exercise. However, recent data suggest that heat interventions may increase isometric strength and endurance performance, without changes in muscle mass, in trained athletes (Scoon et al. 2007; Méline et al. 2021) (Fig. 1f). The impact of passive heat treatment on skeletal muscle mass and properties in healthy participants remains to be clarified. In addition, further studies are needed to establish a stronger consensus on the effects of repeated thermal interventions on sport-specific tasks. For example, little is known about the impact of the regular use of heat intervention on repeated sprint ability in team sports or specific performance in disciplines requiring both endurance and strength qualities (e.g., artistic gymnastics). On the other hand, because of the multifactorial nature of recovery, which also includes psychological components, it is crucial to consider individual preferences (Minett and Costello 2015). Finally, literature does not allow to make definitive statements about the impact of heat interventions on skeletal muscle mass and properties. Further studies are needed in humans to determine the optimal heat dose (e.g., temperature and duration). A dose–response relationship seems to exist, and other stimuli must be considered, such as the hydration level of the participants following the intervention, their training level, the kind of intervention (sauna-bathing, localized heating, hammam, etc.), and fat mass percentage. The studies included in this section and discussing about the effects of repeated heat intervention on adaptations to training and muscle function are summarized in Table 2. Figure 1 details the effects of cold and heat interventions on training adaptations and muscle function.

**Table 2:** Effects of repeated heat intervention on adaptations to training and muscle function

Study and design	Main effects	Exercise	Population and samples	Recovery method	Effects
Scoon et al. 2007	Increased run time to exhaustion and plasma volume	Regular training	Competitive male runners	Sauna	+
				~ 30 min/day; ~ 4 days/week for 3 weeks; ~ 90°C	
Goto et al. 2011	CSA expansion	Only heating	Healthy men	Heat and steam sheets	∅/ +
	Increased MVC of the knee extensors (5.8%) but not the flexors		Vastus lateralis biopsies	8 h/day; 4 days/week for 10 weeks; muscle temperature maintained at 38.3°C	
Tamura et al. 2014	Increased p38 MAPK and p70S6K phosphorylation	ET	Mices (6-week-old males)	Hot chamber	+
	Decreased AMPK and ACC phosphorylation		Plantaris and soleus muscles	30 min/day; 5 days/week for 3 weeks; 40°C	
	Increased mitochondrial enzymes activity and respiratory chain protein content	30 min of treadmill running 5 days/week for 3 weeks at 25 m/min			
Racinais et al. 2017	Improvement of muscle contractile function with an increase of the peak twitch amplitude and the maximal torque production	Only heating	Male participants	Environmental chamber	+
				1 h/day for 11 consecutive days; 48–50 °C at 50% RH	
Hafen et al. 2018	Increase of HSP70, HSP90 and PGC-1alpha expression	Only heating	Healthy sedentary volunteers	Pulsed shortwave diathermy	+
	Increase of mitochondrial electron transport protein complexes I and V expression		Vastus lateralis biopsies	2 h/day for 6 consecutive days; muscle temperature increased by ~ 3.9 °C	
Hung et al. 2018	Decreased shooting performance, postural stability and DHEA-S levels	Regular training	Elite teen archers	HWI	∅/-
	No effect on motor unit recruitment, autonomic system activity or cortisol levels		Saliva	30 min/day; 2 days/week for 2 weeks; 40°C	
Hesketh et al. 2019	Increase in skeletal muscle capillarization and eNOS content	ET	Young sedentary males	Hot chamber	∅/ +
	Increase in skeletal muscle mitochondrial density, GLUT4, or IMTG content in the exercise group only	40–50 min of cycling on a cycle ergometer at an intensity eliciting ~ 65% V̇O_2_peak	Vastus lateralis biopsies	40–50 min/day; 3 days/week for 6 weeks; 40 °C	
Kim et al. 2020	Increase in eNOS content	Only heating	Healthy young adults	Garment perfused with water at ~ 52 °C	+
	Increase the strength of the knee extensors		Vastus lateralis biopsies	90 min/day; 5 days/week; 8 weeks; muscle temperature increased to ~ 37 °C	
Bartolomé et al. 2021	Increase in physical performance (work capacity and muscle flexibility)	Regular training	Semi-professionnal football players	Dry sauna	+
				40 min/day; 3 days/week; 3 weeks, 100 ± 3 °C	
Labidi et al. 2021	No increase in CSA and in strength	Only heating	Active participants	Heat pads	∅
				8 h/day; 5 days/week; 6 weeks; muscle temperature increased by ~ 4.6 °C	
Méline et al. 2021	Increase in maximal isometric strength of the knee extensors	Regular training	Elite short-track speed skaters	HWI	∅/ +
	Increase trend in V̇O_2_max			20 min/day; 4 days/week for 4 weeks; 40.3 °C	
Cui et al. 2022	Decreased resting sympathetic activity and heart rate	Only heating	Healthy older volunteers	HWI	+
				30 min/day; 5 days/week for 4 weeks; ∼40 °C	
Marchant et al. 2022	Increase in mitochondrial respiratory capacity	RT (4 × 4 min of single-leg extension at 80% of work rate max with 4 min of active recovery at 20% of work rate max)	Sedentary young subjects	Short-wave diathermy	∅/ +
	Fat oxidation capacity, citrate synthase activity and exercise capacity increased in the exercise group only		Quadriceps femoris biopsies	60 min/day; 3 days/week for 6 weeks; muscle temperature elevated at 39.5°C	
Sitkowski et al. 2022	No additional effects on hematological variables and on exercise capacity	ET	Physical education students	Sauna bathing	∅
		60 min stationary cycling	Blood samples	30 min/day; 3 days/week for 4 weeks; 89 ± 3 °C, 10 ± 2% RH	
Horgan et al. 2023	No increase in lean muscle mass and in resistance performance	Heat-RT	Rugby players	HWI	∅
				15min/day; 2 days/week por 4 weeks; 39.3°C	
Ihsan et al. 2023	No increase in gastrocnemius aerobic function	Only heating	Physically active participants	Heat pads	∅
				8 h/day; 5 days/week for 6 weeks; muscle temperature elevated at 37.6°C	

*ACC* acetyl-coenzymeA carboxylase, *CS* citrate synthase, *CSA* cross-sectional area, *DHEA-S* dehydroepiandrosterone sulfate, *eNOS* endothelial nitric oxide synthase, *ET* endurance training, *GLUT4* glucose transporter type 4, *HAD* 3-hydroxyacyl coenzymeA dehydrogenase, *HSP* heat shock protein, *HWI* hot water immersion, *IGF-1* insulin-like growth factor 1, *IGF2R* insulin-like growth factor2 receptor, *IMTG* intramuscular triglyceride, *[La*^*−*^*]* blood lactate concentration, *MVC* maximal voluntary contraction, *p70S6K* ribosomal protein70 S6 kinase, *PGC-1alpha* peroxisome proliferator-activated receptor-gamma coactivator-1alpha, *PHF20* plant homeodomain finger protein 2, *RT* resistance training, *TNIK* Traf2- and Nck-interacting kinase, *TFDP2* transcription factor, *Dp-2/E2F* dimerization partner 2, *TTN* titin, *UQCRB* ubiquinol-cytochrome c reductase binding protein, *V̇O*_*2*_*peak* peak oxygen uptake

### Exercise and thermal interventions alter epigenetic factors: current evidence and perspectives

Recent investigations have revealed that endurance exercise provoked global and gene-specific changes in the methylation levels in skeletal muscle biopsies of healthy sedentary men (Barrès et al. 2012). Concretely, the authors observed reduced methylation at the promoters of several genes involved in cellular metabolism: PGC-1alpha, PDK4 (pyruvate dehydrogenase kinase 4), and PPAR-delta (peroxisome proliferator-activated receptor-delta), along with increased mRNA abundance. This suggests that changes in the methylation level of these genes are correlated with mRNA expression. Importantly, these results depended on exercise intensity, with stronger hypomethylation and expression modifications after high-intensity exercise. Training intervention also impacts DNA methylation during endurance exercise, as shown by a study conducted on humans with unilateral endurance tasks (Lindholm et al. 2014). However, these modifications mostly concerned enhancers, gene bodies and intergenic regions. These data suggest that while promoters may be involved in quick adjustments, other regions are likely involved in long-term adaptations to exercise training. In this sense, twelve weeks of resistance and high-intensity interval training led to minor changes in the methylation of DNA promoter regions (Robinson et al. 2017). A recent study conducted in our laboratory showed that global methylation increases after a single sprint interval training (Solsona et al. 2024). Furthermore, the methylation of an enhancer of the gene-inducible NO synthase increased after exercise, suggesting that exercise is a potent stimulus to induce epigenetic modifications. Importantly, the magnitude of muscle deoxygenation during exercise was associated with the changes in DNA methylation of both the promoter and the enhancer of this gene (the relationship was negative for the enhancer). Furthermore, average heart rate was also correlated with the modifications of the promoter methylation. These results offer insight into the potential link between the stress induced by a sprint interval session and alterations in methylation patterns. Further research is necessary to determine if these modifications influence gene expression.

The relationship between resistance exercise and DNA methylation has also been explored, although the number of studies in this domain remains limited. Nonetheless, these studies have provided valuable insights into the fluctuations in DNA methylation and their connection to gene expression (Seaborne and Sharples 2020). For instance, research has shown that resistance training elicits differential methylation and messenger RNA expression changes of growth factors such as growth hormone-releasing hormone and fibroblast growth factor-1 in the leukocytes of young men (Denham et al. 2016). On the other hand, acute resistance exercise can modulate global methylation as evidenced by the hypomethylation of long interspersed nuclear element-1 in resistance-trained, but not in sedentary subjects (Bagley et al. 2020). Interestingly, it was found that metabolic genes such as glycerol-3-phosphate acyltransferase (GPAM) and sterol regulatory element binding transcription factor 2 (SREBF2) exhibited hypermethylation in accustomed participants. At the same time, the methylation levels decreased for SREBF2 in the inexperienced group (Bagley et al. 2020). Thus, opposite responses were obtained for SREBF2 depending on the training status. These contrasting findings could be attributed to the fact that resistance training represents a metabolic stress for sedentary people, whereas trained subjects may experience less disruption to metabolic homeostasis. Given the inverse association between gene methylation and expression levels (Cortés-Mancera et al. 2022), this result suggests that SREBF2 was expressed in the unexperienced group and inactivated in resistance-trained individuals, even though SREBF2 mRNA levels were not measured in the study. Therefore, epigenetic responses may largely depend on the fitness status, probably because it can modulate the stress response to a given type of exercise. Surprisingly, the methylation levels of genes associated with inflammation (IL-6 and TNF-alpha) or hypertrophy (MTOR and Akt) were not affected in either group. Furthermore, in the context of exercise training, epigenetic modifications also rely on the nutritional status. In a crossover study, trained cyclists performed a high-intensity interval exercise (8 × 5 min at 82.5% of peak power output) in the evening before being split into two groups: fasted and fed (Lane et al. 2015). The next morning, they all completed 2 h of cycling at 50% of peak power output before being fed with a standardized meal. The results of the study show that 4 h post-exercise, the promoter of PPAR-delta exhibited hypermethylation in the overnight fasting condition only.

It has been established that environmental conditions affect cellular function through epigenetic mechanisms. Importantly, different heat exposure durations have diverse effects on the epigenome (Malabarba et al. 2021). Hot conditions regulate miRNAs, single interfering RNAs, long non-coding RNAs, histone variants, chaperones and covalent modifications, chromatin remodeling and DNA methylation in plants (Liu et al. 2015). These results are of paramount importance because they establish a wide range of epigenetic modifications in response to heat exposure. Similarly, miRNA-297 was upregulated during the heat acclimatization period in rats (Tetievsky et al. 2014), showing that alterations in the epigenome occur in mammals with thermal interventions. In cultured mouse skeletal myoblasts, thermal stress also influences epigenetics, as shown in a study that examined the impact of different temperatures on histone modifications (Sajjanar et al. 2019). In this study, the relative expression of the histone acetyltransferase Gcn5 and histone H4-acetylation increased at 41 °C. In high-temperature conditions, the relative mRNA level of the de novo DNA methyltransferase 3A (DNMT3A) increased, while it decreased in cold conditions. Contrastingly, the methyltransferase responsible for the maintenance of methylation (DNMT1) increased in the cold conditions (Sajjanar et al. 2019). Global DNA methylation responded differently in the heat versus cold conditions, with a higher amount of 5-methylcytosine at 39 °C compared to 35 °C. Interestingly, heat stress reduced maximal respiration and spare respiratory capacity, suggesting impaired mitochondrial function in C2C12 myoblasts. Conversely, cold stress stimulated glycolysis as a thermogenic response to meet energy requirements. Lastly, glycolytic capacity increased in all conditions compared to the control. Taken together, these results indicate an interplay between energy metabolism and epigenetics during cell stress response (Sajjanar et al. 2019). As acetyl and methyl groups are covalent modifications, they appear to be thermodynamically stable (Delatte and Fuks 2013). Thus, although the mechanisms behind the modulation of epigenetic modifications are not fully understood, these data seem to indicate that hot and cold conditions differently affect enzymes that regulate epigenetic marks and the subsequent adaptations to stress.

The effects of thermal interventions on ten-eleven translocation (TET) proteins (*i.e.*, DNA demethylases) should be assessed for a deeper understanding of the regulation of DNA methylation. Importantly, it has been shown that physical activity prevents the decreased expression of TET proteins, as shown in the hippocampus of aged mice (Jessop and Toledo-Rodriguez 2018). Consequently, the promoter region of miRNA-137 was rich in 5-hydroxymethylcytosine, suggesting enhanced neurogenesis and improved memory. However, this result was not found in the hypothalamus, indicating distinct regulation processes according to the cerebral zones. On the opposite, exercise training hypermethylated genes related to hypertension while TET1 expression decreased in hypertensive rats, contributing to enhanced mesenteric arterial function and vasodilation (Zhang et al. 2020). Another study showed that heat-induced global hypomethylation in microspores and the amount of differentially methylated genes were temperature-dependent (Li et al. 2016), evidencing a dose–response relationship. Correspondingly, thermal stress reduced the levels of DNA methylation in corals, which hypothetically leads to transcriptional plasticity (Dimond and Roberts 2016). This means that heat exposure could open a window of opportunity for enhancing training adaptations. Another study assessed the impact of 21 days of heat exposure on epigenetics in pigs. The data showed that constant heat stress induced global hypermethylation, but several genes involved in muscle growth and development were hypomethylated. Myosin heavy chain 11, implicated in muscle fiber properties, was identified among the hypomethylated genes. Additionally, genes encoding glycolytic enzymes also displayed differential methylation, leading to increased protein expression. For example, PDK3 was hypomethylated under constant heat conditions, resulting in increased expression. PDK3 plays a role in inhibiting the conversion of pyruvate to acetyl-coenzyme A, thereby inhibiting aerobic processes in skeletal muscle (Hao et al. 2016). Taken together, these data strongly suggest that thermal interventions affects the epigenome.

Overall, the effects of exercise on epigenetics need further attention, especially in humans and in combination with thermal interventions. The influence of epigenetic modifications on the expression of specific genes should be a growing research axis in the next years. Indeed, it represents an essential perspective for both athletes and patients to better understand the effectiveness of exercise prescriptions on skeletal muscle adaptations. Of note, epigenetic responses are tissue- and species-specific (He et al. 2011; Weyrich et al. 2020), and consequently, studies on the effects of heat exposure on humans are needed, notably in skeletal muscle.

## Thermal interventions and skeletal muscle regeneration

Despite its notable impact on pain perception, cold intervention appears to hinder the process of muscle regeneration in numerous animal studies. Research on rats and mice revealed that cryotherapy delays recovery after injury (Takagi et al. 2011; Miyakawa et al. 2020; Kawashima et al. 2021; Miyazaki et al. 2023). Cryotherapy can reduce the inflammatory response following skeletal muscle injury (i.e., freezing injury of the tibialis anterior in rats) by decreasing the infiltration of pro-inflammatory macrophages and the concentration of several inflammatory markers (Vieira Ramos et al. 2016). As evidenced by another study in rats, a twenty-minute cooling intervention immediately succeeding trauma delayed skeletal muscle regeneration (Takagi et al. 2011). Specifically, during the primary reaction of degeneration and the early stages of regeneration, the authors observed a delayed macrophage migration to the injured site. The proportion of centrally nucleated fibers was higher in the icing group 14 days after injury, suggesting a slower regeneration. Furthermore, the CSA of the regenerating fibers was larger in the non-icing group 28 days post-trauma. Notably, collagen deposition was significantly higher in the icing group both at 14 and 28 days after injury. Still in the context of muscle contusion injuries in rats, icing has been associated with delayed neutrophil and macrophage infiltration (Singh et al. 2017). The authors showed a reduced/delayed infiltration of macrophages, reduced expression of angiogenic factors, and lower vessel volume for the cold group. Moreover, it was recently found that icing decreases M1 macrophage accumulation after skeletal muscle injury in rats (i.e., electrically stimulated eccentric model), leading to an incorrect degradation of injured muscle fibers (Kawashima et al. 2021; Miyazaki et al. 2023). As a result, delayed and impaired muscle regeneration was observed.

Hence, the successive phases of muscle degeneration and short-term inflammation appear essential for subsequent proper regeneration (Takagi et al. 2011; Miyakawa et al. 2020). Upon muscle injury, calcium release may activate the calpain proteases, initiating the removal of myofibrillar fragments before proteasomes can degrade these damaged structures (Bartoli and Richard 2005). However, temperature is an important regulator of the enzymatic activity of calpains (Pomponio and Ertbjerg 2012). Additionally, inflammation might play a crucial signaling role in the initiation of the regenerative process. Cold intervention by blunting inflammation disrupts the normal progression of recovery and alters the regeneration cascade. However, a recent study in rats introduces a contrasting perspective. In comparison to previous studies, Nagata et al. used a milder crush injury model inducing a limited muscle injury with a myofiber necrosis fraction of approximately 4% of the myofibers. Icing treatment (3 sets of 30 min immediately after injury, each with 90-min intervals, and 24 h and 48 h after), decreased the accumulation of inducible NO synthase-expressing macrophages and the percentage of necrotic cells, and increased the number of activated satellite cells and CSA, suggesting an improvement in skeletal muscle regeneration (Nagata et al. 2023). Interestingly, the authors suggested that icing could be an appropriate solution for improving regeneration from limited muscle damage, but should be avoided in case of large-scale muscle necrosis.

The extrapolation of the results of these studies to humans presents limitations. Indeed, only one of these studies used an eccentric-damaging model, prompting whether the damage induced by freezing or muscle crush injuries is comparable to EIMD. Moreover, due to the smaller size of rat legs, applying ice for 30 min provides a severe cooling effect, whereas the same dose in humans may not result in comparable reductions in muscle temperature (Bleakley et al. 2012). In humans, cryotherapy decreases the expression of blood markers of skeletal muscle inflammation (*i.e.,* interleukin-1 and C-reactive protein), as well as oxidative stress markers (H_2_O_2_ and NO) after a 14-day training camp (Zembron-Lacny et al. 2020). It has been suggested that the attenuation of these indirect markers of muscle stress might delay skeletal muscle regeneration (Zembron-Lacny et al. 2020). Indeed, a decrease in inflammation and oxidative stress might generate dysfunctional autophagy in muscle fibers, leading to an incomplete degradation of damaged organelles, and resulting in a delayed or impaired regeneration of skeletal muscle (Sandri et al. 2013; Sanchez et al. 2019; Romanello and Sandri 2021). However, to date, there are no studies that have focused on the impact of cryotherapy following a skeletal muscle injury in humans, even if it is a commonly used practice in sports traumatology (Frery et al. 2023).

**Fig. 2:**
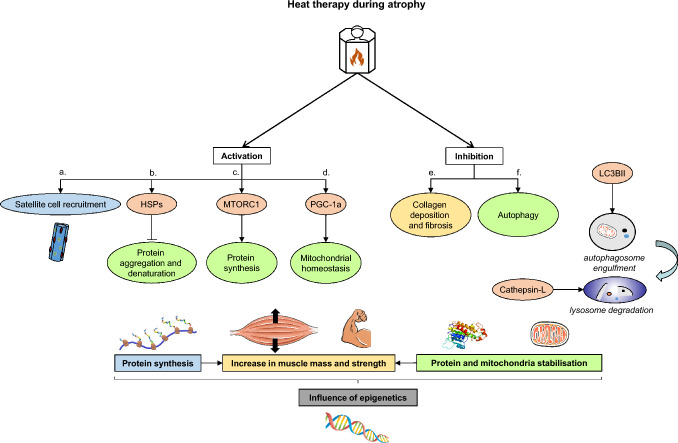
Muscle regeneration with heat intervention during atrophy. Heat intervention promotes protein synthesis through several mechanisms: **a** promotion of satellite cell proliferation and differentiation, **b** induction of HSPs, and **c** modulation of the MTORC1 pathway. Heat intervention enhances mitochondria homeostasis via the PGC-1alpha pathway (**d**), and limits skeletal muscle fibrosis, collagen deposition and protein aggregation (**e**). Additionally, heat intervention reduces the activity of the autophagosome-lysosome system (*Cathepsin* L.), including mitophagy (**f**). However, most of the studies are conducted in non-human models (i.e., rat and mouse models) and translation between species remains challenging. In this sense, more research in humans is needed to establish the optimal protocol for incorporating heat intervention into therapeutic approaches. Future research is necessary to further understand the involvement of epigenetic modifications on the effects of heat intervention in skeletal muscle regeneration. These adaptations collectively contribute to the increases in skeletal muscle mass and isometric strength during muscle injury

Data on the effects of heat intervention during skeletal muscle regeneration have also been explored. In a recent study examining the autophagic response during atrophy in rodents with sectioned soleus and plantaris tendons, the repeated application of heat mitigated the reduction in fiber CSA (Hirunsai and Srikuea 2021). This study demonstrated the beneficial effect of heat application using thermal blankets (40.5–41.5 °C, 30 min/day for 7 days) on the autophagic pathway in rats, particularly in the context of tenotomy-induced muscle atrophy. Moreover, the observed atrophy was linked to an elevation in autophagic markers. Of note, autophagy represents an important system in the degradation and renewal of cellular constituents (Sanchez et al. 2019; Botella et al. 2023). During stressful situations such as exercise, heat exposure, hypoxia or fasting, double-membrane vesicles called autophagosomes capture cytoplasmic components, including lipids, proteins, and organelles such as mitochondria and ribosomes to limit unnecessary energy expenditure (Sanchez et al. 2019). These vesicles are subsequently engulfed by lysosomes and are degraded along with their contents. Again, heat intervention effectively targets and limits autophagic processes at eight and fourteen days of immobilization, suggesting its role in preserving cellular components (Hirunsai and Srikuea 2021) (Fig. 2f). Heat treatment (thermal blankets, 30 min/day, 40.5–41.5 °C, 7 days) was found to reduce the expression of the protein Cathepsin L in the atrophied soleus muscle of rats, suggesting a decrease in lysosomal activity (14 days post-tenotomy). Consistently, heat intervention significantly limits the loss of muscle mass during atrophy in rats (Goto et al. 2004; Hirunsai and Srikuea 2021). Moreover, heat treatment (30 min/day, 42 °C, 6 weeks) reduces muscle atrophy (evidenced by CSA measurements) and increases markers of protein synthesis (Akt, mTOR, heat-shock protein (HSP) 70) in diabetic rats (AlSabagh et al. 2022) (Fig. 2 c.). Heat pre-conditioning (20 min, 43 °C, 48 h before exercise) also increased HSPs expression and attenuated the increases in CK plasma concentration induced by downhill running in rats (Touchberry et al. 2012). Heating skeletal muscle can thus prevent the overactivation of this catabolic system in oxidative and glycolytic muscles. Additionally, heat exposure (thermal blankets, 30 min/day, 40.5–41.5 °C, 7 days) reduces necrosis and decreases the expression of the apoptotic protein TNF-alpha in tenotomized soleus muscles in rats (Hirunsai and Srikuea 2020). During atrophy, the infiltration of macrophages was reduced with heat interventions. Heat intervention can also limit fibrosis, decreasing collagen deposition (Fig. 2e) in injured rat soleus muscle (bupivacaine injection), whereas cold stress has the opposite effect (Shibaguchi et al. 2016). A recent review includes heat intervention as a potential therapeutic strategy for skeletal muscle atrophy (Huang et al. 2022). Specifically, heat treatment upregulates HSP72, HSP25 and downregulates REDD1 (regulated in development and DNA damage responses 1), preventing muscle wasting (Nonaka et al. 2015; Tsuchida et al. 2017). Moreover, in rats with diabetes-induced atrophy, heat intervention attenuates the decrease in fiber CSA (Nonaka et al. 2015). Finally, a recent meta-analysis concludes that passive heat treatment can act as an exercise mimetic and can be used as an alternative to exercise to increase muscle mass and strength and attenuate subsequent atrophy (Rodrigues et al. 2020b). However, unlike physical exercise, heat treatment only provokes small changes in tissue metabolism (Kaluhiokalani et al. 2024). Localised passive heat therapy, applied intermittently over six weeks, induces similar gains in resistance artery function (i.e., peak blood flow) at rest compared to exercise training, but smaller gains during a knee extension test (Kaluhiokalani et al. 2024). Moreover, even if the increase in endothelial nitric oxide synthase expression was similar between the two conditions, only exercise training enhanced angiogenesis (Kaluhiokalani et al. 2024). Additionally, unlike whole-body hyperthermia, local hyperthermia does not increase ventilation, systemic aerobic metabolism and left ventricular systolic and diastolic functions, even if leg blood flow and cardiac output were increased (Watanabe et al. 2024). The authors suggest that local hyperthermia, with leg tissue temperature ≤ 38 °C, is not a sufficient stimulus to activate metabolism and central cardiorespiratory-mediated mechanisms. Hence, passive local heat treatment seems to induce fewer adaptations compared to exercise. Therefore, heat exposure should not be considered a substitute for exercise training but rather a complementary intervention.

A recent study examined the effects of passive muscle heating (hot pack, 42 °C, 20 min) on skeletal muscle regeneration after a crush injury on the *extensor digitorum longus* muscle in rats (Takeuchi et al. 2014). A significant increase in ED1 macrophages was observed in the heat group, occurring earlier than in the non-heat group. Moreover, there was a significant increase of Pax-7 (paired box protein 7) positive satellite cells at 4 and 6 days after the crush injury in the heat group (Fig. 2a). Hence, the authors suggested that acceleration of the degeneration process (faster increase in ED1 macrophages) by heating could be a solution to promote regeneration. Similar results were reported in another study, where heating stimulated satellite cell proliferation and protein synthesis during the regeneration of injured skeletal muscle in rats (Kojima et al. 2007). A recent study examined the effects of heat intervention (20 min, 40 °C, after injury) on muscle fiber regeneration after muscle injury (*i.e.*, injection of cardiotoxin) in rats (El-Sheikh et al. 2022). In the heating group, the regenerating myotubes (defined as those with two or more central nuclei) were observed at three days post-injury, whereas in the non-heating group, they were observed at seven days post-injury. Hence, heat treatment hastens skeletal muscle regeneration. Moreover, passive muscle heating (hot packs, 42 °C, 20 min) accelerates the increase in myoblast determination protein (MyoD) and myogenin protein expression during the regeneration process in rats (Hatade et al. 2014). These proteins play pivotal roles in myogenesis during embryonic/neonatal stages (Weintraub 1993) and muscle regeneration (Marsh et al. 1997). Additionally, applying heating shortly after muscle injury can accelerate the proliferation and differentiation of myogenic cells, promoting faster muscle repair (Hatade et al. 2014).

It is well established that immobilization leads to a significant loss of muscle mass, strength and function in both elderly patients and injured athletes (Tipton 2015; Rommersbach et al. 2020). Furthermore, it has been shown that, in mice, heat exposures (40 °C, 30 min/day, 7 days) limit mitochondrial loss in denervated muscle, thus reducing the subsequent atrophy (Tamura et al. 2015). The interest in the impact of heat treatment on skeletal muscle regeneration in humans is increasing. A recent study examined the effects of 2-h daily heating (short-wave pulsed diathermy) during a 10-day immobilization period in humans. Heat intervention maintained mitochondrial function, attenuated atrophy, and increased the expression of PGC-1alpha (Hafen et al. 2019) (Fig. 2d). Furthermore, a significant increase in HSP70 and 90 protein content was found after immobilization in the group using heat intervention only (Hafen et al. 2019) (Fig. 2b). HSPs play a chaperoning role: they prevent the loss of function of other proteins by assisting their correct folding/refolding and preventing protein denaturation and aggregation (Dahiya and Buchner 2019) (Fig. 2b). They can be activated by high temperature (heat stress), hypoxia (Garbuz 2017) and exercise (Archer et al. 2018). Studies in rats indicate that HSPs exhibit important antioxidant properties and contribute to preserving muscle mass during immobilization periods (Selsby and Dodd 2005; Selsby et al. 2007). Among the HSP family, HSP70 has been shown to play a major role in the integrity of the muscle cell and its regeneration after a traumatic event in mice (Senf 2013). With a deficiency of HSP70, there is an increase in inflammation (macrophage infiltration in muscle fibers) and necrosis (Senf et al. 2013). Precisely, after injury (i.e., injection of cardiotoxin), average fiber CSA was lower and the number of regenerating fibers containing centralized nuclei was higher in HSP70-deficient mice. Logically, a decreased muscle regeneration was also observed. Finally, a recent study examined the effects of whole-body heat intervention during two weeks of immobilization of a single lower leg (Labidi et al. 2024). The authors found that heat treatment prevented the decrease in maximal isometric strength of the plantar flexors and the decrease in the soleus CSA. Additionally, the FOXOs and NFκB (nuclear factor-kappa B) signaling pathways, which are involved in skeletal muscle atrophy, showed reduced activation with heat treatment. Thus, heat exposure appears as a promising intervention for mitigating the negative effects of immobilization in humans. Furthermore, DNA methylation patterns are also altered by disuse atrophy. Fisher and coworkers showed that, by paralyzing the tibialis anterior muscle of rats, the promoter methylation of genes related to atrophy (i.e., muscle ring-finger protein-1 (MuRF1) and MAFbx/Atrogin-1) decreases (Fisher et al. 2017). Therefore, it is crucial to investigate the effects of single and repeated exercise and thermal interventions on the epigenome after immobilization.

In summary, in animal models, cold intervention attenuates the oxi-inflammatory response following skeletal muscle injury, potentially leading to dysfunctional autophagy, which generates an incomplete degradation of organelles in the muscle cell in the early recovery phase. This mechanism might partly explain the observed delay in skeletal muscle regeneration with cold application. Conversely, heat intervention appears to be a promising treatment for treating muscle injury, promote regeneration, and preserve muscle mass and function. Considering the crucial role of muscles in overall physical function, these benefits could potentially translate into improved quality of life for individuals with cardiovascular disease (Monroe et al. 2020) or sarcopenic populations (Sanchez and Solsona 2021). Enhanced muscle health and function can lead to increased mobility, reduced fatigue, and improved ability to perform daily activities. This, in turn, can contribute to a higher level of independence and overall well-being. Furthermore, the positive impact of heat treatment on muscle health may indirectly contribute to increased longevity. As cardiovascular disease and age-related muscle loss are associated with higher mortality rates, interventions that can mitigate these conditions may extend life expectancy. However, most of the studies are performed in non-human models (i.e., rat and mouse models) and translation between species remains challenging. In this sense, more research in humans is needed to establish the optimal protocol for incorporating heat intervention into therapeutic approaches. The studies included in this section and treating about the effects of thermal interventions on skeletal muscle regeneration are summarized in Tables 3 (cold intervention) and 4 (heat intervention). Figure 2 details the effects of heat intervention on skeletal muscle regeneration during atrophy.

**Table 3:** Effects of cold intervention on skeletal muscle regeneration

Study and design	Main effects	Injury or immobilization model	Population and samples	Recovery method	Effects
Takagi et al. 2011	Delayed macrophage migration	Crush injury (30 s)	Rats	Ice pack	–
	Lower increase in CSA and high proportion of centrally nucleated fibers		Extensor Digitorum Longus	20 min; 0.3 ~ 1.3 °C	
Vieira Ramos et al. 2016	Decreased inflammatory processes (macrophage percentage and mRNA levels of inflammatory markers)	Freezing injury (2 × 10 s)	Rats (three-month-old males)	Ice pack	+
	No alteration of regeneration process (injury area, desmin and myoD expression)		Tibialis anterior	3 × 30 min; immediately, 24 h and 48 h after injury; muscle surface temperature decreased by 17 °C	
Shibaguchi et al. 2016	Increase the development of fibrosis	Bupivacaine injection	Rats (8-week-old males)	Ice pack	–
	Delay in the timing of disappearance of TGF-β		Soleus	20 min; 0 °C	
Singh et al. 2017	Delayed neutrophil and macrophage infiltration	Contusion injury	Rats (12-week-old males)	Ice pack	∅/–
	Higher percentage of immature myofibers		Biceps Femoris	20 min; 0.3 ~ 1.3 °C	
	No differences in CSA and capillary density				
Miyakawa et al. 2020	Inhibition of the accumulation of macrophages	Crush injury (30 s)	Rats (8-week-old males)	Ice pack	–
	Fewer neutrophils and MCP-1 + cells		Extensor Digitorum Longus	20 min; 0.3 ~ 1.3 °C	
Kawashima et al. 2021	Persistence of necrotic muscle debris	EIMD	Mice (13–14 weeks old)	Ice pack	–
	Delayed macrophage M1 infiltration		Gastrocnemius		
	Delay in the emergence of Pax7 + myogenic cells			3 × 30 min; immediately, 24 h and 48 h after injury; muscle surface temperature decreased by 17 °C	
Miyazaki et al. 2023	Decrease in the accumulation of M1 macrophages	Crush injury	Rats	Icing	–
	Decrease in TNF-α expression				
	Decrease in the number of myogenic precursor and the size of centrally nucleated regenerating myofibers				
Nagata et al. 2023	Decreased the accumulation of inducible nitric oxide synthase-expressing macrophages and the percentage of necrotic cells	Milder crush injury (30 s)	Rats (8-week-old males)	Ice pack	+
	Increased the number of activated satellite cells and cross-sectional area		Extensor digitorum longus	3 × 30 min; immediately, 24 h and 48 h after injury; muscle surface temperature decreased by 16 °C	

*EIMD* eccentric contraction-induced muscle damage, *MCP1* monocyte chemoattractant protein 1, *Pax7* paired box protein 7, *TGF-β* transforming growth factor beta, *TNF-α* tumor necrosis factor α

**Table 4:** Effects of heat intervention on skeletal muscle regeneration

Study and design	Main effects	Injury or immobilization model	Population and samples	Recovery method	Effects
Goto et al. 2004	Stimulation of protein synthesis	Hindlimb suspension (5 days)	Rats (10-week-old males)	Incubator	+
	Increase in HSP72 expression		Soleus	60 min; 41 °C	
Selsby and Dodd 2005	Increase in HSP25 and HSP72 expression	Bilateral immobilization in the plantarflexed position (8 days)	Rats (males)	Heat chamber	+
	Reduction in oxidative damage		Soleus	30 min; every 2 days; 8 days; core temperature maintained at 41–41.5 °C	
	Lower decrease in muscle mass				
Kojima et al. 2007	Stimulation of satellite cell proliferation and protein synthesis	Cardiotoxin injection	Rats (7-week-old males)	Heat chamber	+
			Tibialis anterior	60 min; 24 h before or immediately after injury; 41 °C	
Selsby et al. 2007	Potentiated muscle regrowth and reduced oxidant damage	Bilateral immobilization in the plantarflexed position (7 days)	Male Rats	Heat chamber	+
	Increase of HSP25, HSP32, and HSP72 protein content		Soleus	30 min; every 2 days; 7 days; core temperature maintained at 41–41.5 °C	
Touchberry et al. 2012	Increase in HSP72 expression and total protein concentration	EIMD (Downhill running)	Rats (10-week-old males)	HWI	+
	Decrease in CK plasma levels		Soleus	20 min; 48 h prior to exercise; 43 °C	
Hatade et al. 2014	Faster increase of MyoD and myogenin protein expression	Crush injury (30 s)	Rats (8-week-old males)	Hot pack with water	+
			Extensor digitorum longus	20 min; 5 min after injury; 42 °C	
Takeuchi et al. 2014	Increase of ED1 macrophages earlier	Crush injury (30 s)	Rats (8-week-old males)	Hot pack with water	+
	Increase of Pax-7 positive satellite cells at 4 and 6 days after the crush injury		Extensor digitorum longus	20 min; 5 min after injury; 42 °C	
	Increase in CSA				
Nonaka et al. 2015	Increase in HSP25 and HSP72 expression	Atrophy induced by diabetes (injection of streptozotocin)	Rats (12-week-old males)	HWI	+
	Lower decrease in CSA		Extensor Digitorum Longus	30 min; 7 days after injection of streptozotocin and 5 days/week for 3 weeks; 42 °C	
Tamura et al. 2015	Limit mitochondrial and muscle mass loss	Sciatic nerve transection	Mice (8-week-old males)	Heat chamber	+
			Gastrocnemius	30 min/day for 1 week; 40 °C	
Shibaguchi et al. 2016	Decrease in fibrosis	Bupivacaine injection	Rats (8-week-old males)	HWI	+
	Increase in muscle mass, protein content and muscle fiber size		Soleus	30 min; 48 h after injury and everyday for 12 days; 42 °C	
Hafen et al. 2019	Maintain mitochondrial function and increase the expression of PGC-1alpha	Immobilization of the left leg (10 days)	Healthy volunteers	Short-wave pulsed diathermy	+
	Increase in HSP70 and HSP90		Vastus lateralis	120 min everyday for 10 days; intramuscular temperature increased by 4.2 ± 0.29 °C	
	Lower decrease in CSA				
Hirunsai and Srikuea 2020	Reduction of necrotic fibers, M1 macrophage invasion and TNFα protein expression	Tenotomy (Achilles tendon ablation)	Rats (10-week-old males)	Thermal blankets	+
	Increase of M2 macropage accumulation		Soleus and plantaris	24 h before tenotomy and 3 days/week for 2 weeks; core temperature maintained at 40.5–41.5 °C	
Hirunsai and Srikuea 2021	Lower decrease in CSA	Tenotomy (Achilles tendon transection)	Rats (10-week-old males)	Thermal blankets	+
	Decrease in autophagy-lysosomal signaling pathway		Soleus and plantaris	30 min/day for 7 days; core temperature maintained at 40.5–41.5 °C	
AlSabagh et al. 2022	Lower decrase in CSA	Atrophy induced by diabetes	Rats (3-month-old males)	Heat chamber	+
	Decrease in muscle atrophy markers (CD68, KLF, MAFbx)			30 min/day; everyday for 6 weeks; 42 °C, 15.0 ± 2.0% RH	
	Increase in muscle hypertrophy markers (AKT, mTOR, HSP70)				
El-Sheikh et al. 2022	Earlier increase of regenerating myotubes	Cardiotoxin injection	Rats	How water bottle	+
			Tibialis anterior	20 min; 5 min after injury; 40 °C	
Labidi et al. 2024	Lower decrease in maximal isometric strength and CSA	Single-lower leg immobilization (2 weeks)	Male participants	Whole-body heat therapy	+
	Lower activation of FoxO and NFκB signaling pathways		Soleus and gastrocnemius		

*CK* creatine kinase, *CD68* cluster of differenciation 68, *KLF* Krüppel-like family of transcription factors, *HWI* hot water bath, *FoxO* forkhead box O, *MAFBX* muscle atrophy F-box, *mTOR* mechanistic target of rapamycin, *MyoD* myoblast determination protein, *NFκB* nuclear factor-kappa B

## Conclusions

In conclusion, recent literature highlighted that thermal interventions can interfere with cellular adaptations involved in gains of physical aptitudes. Recent research showed that repeated post-resistance-exercise cold interventions inhibit several gains in hypertrophy, isometric and endurance strength. From a molecular viewpoint, the regular use of cryotherapy reduces oxi-inflammatory responses, downregulates the MTORC1 pathway, ribosome biogenesis and regeneration. On the other hand, heat intervention can stimulate protein synthesis, however, to date, no supplementary effect has been observed on skeletal muscle mass in trained humans. However, increases in isometric strength, blood volume and endurance performance have been observed in sub-elite and elite athletes. Importantly, heat intervention promotes muscle regeneration in the context of atrophy. In humans, heat treatment increases the expression of HSPs and PGC-1alpha, and decreases the autophagy pathway, which leads to preserved mitochondrial function and attenuated atrophy during immobilization. Therefore, heat intervention may represent an important tool for reducing atrophy and maintaining mitochondrial function during aging or immobilization. Furthermore, significant progress has been made in understanding the mechanisms underlying the effects of introducing heat treatment in the context of exercise and rehabilitation. In this way, epigenetic modifications such as DNA methylation and histone modifications might play a key role in the physiological adaptations induced by exercise and thermal interventions. Importantly, as blood markers represent an indirect measure, future studies must prioritize muscle tissue to evaluate the effects of thermal interventions. Further research should focus on determining the optimal timing and the optimal “stress” of these interventions to promote gains in performance and to develop complementary therapeutic approaches. This research topic is essential to enhance skeletal muscle adaptations and to mitigate muscle wasting in various pathological states and during immobilization periods.

## Abbreviations

CSA: Cross-sectional area
CWI: Cold-water immersion
CK: Creatine kinase
DNMT: DNA methyltransferase
DOMS: Delayed-onset muscle soreness
EIMD: Exercise-induced muscle damage
FOXOs: Forkhead box class O proteins
H_2_O_2_: Hydrogen peroxide
HSP: Heat shock protein
HWI: Hot-water immersion
IL-6: Interleukin-6
miRNAs: MicroRNAs
MAFbx: Muscle atrophy F-box gene
MTORC1: Mammalian/mechanistic target of rapamycin complex 1
NO: Nitric oxide
NOS: Nitrogen oxide species
PDK: Pyruvate dehydrogenase kinase
PGC-1alpha: Peroxisome proliferator-activated receptor-gamma coactivator-1alpha
PPAR-delta: Peroxisome proliferator-activated receptor-delta
ROS: Reactive oxygen species
SREBF2: Sterol regulatory element binding transcription factor 2
TET: Ten–eleven translocation
TNF-alpha: Tumor necrosis factor-alpha
${\dot{V}O}_{2}max$: Maximal oxygen consumption
WBC: Whole-body cryotherapy
